# Sodium fluoride (NaF) causes toxic effects on splenic development in mice

**DOI:** 10.18632/oncotarget.13971

**Published:** 2016-12-16

**Authors:** Ping Kuang, Huidan Deng, Hengmin Cui, Lian Chen, Jing Fang, Zhicai Zuo, Junliang Deng, Xun Wang, Ling Zhao

**Affiliations:** ^1^ College of Veterinary Medicine, Sichuan Agricultural University, Ya’an 625014, China; ^2^ Key Laboratory of Animal Diseases and Environmental Hazards of Sichuan Province, Sichuan Agricultural University, Ya’an 625014, China

**Keywords:** NaF, growth index, T and B-cell, cytokine, cell cycle

## Abstract

At present, very limited studies focus on the toxic effect of sodium fluoride (NaF) on splenic development of human and animals *in vivo*. This study was firstly designed to evaluate the toxic effects of NaF on the splenic development of mice in vivo by observing histopathological lesions, changes of splenic growth index (GI), T and B cells, immunoglobulin A (IgA), immunoglobulin G (IgG) and immunoglobulin M (IgM) contents, cytokine protein expression levels, and cell cycle and cyclins/cdks protein expression levels using the methods of pathology, flow cytometry (FCM), western blot (WB), and enzyme-linked immunosorbent assay (ELISA). A total of 240 ICR mice were equally allocated into four groups with intragastric administration of distilled water in the control group and 12, 24, 48 mg/kg NaF solution in the experimental groups for 42 days. The results showed that NaF in 12 mg/kg and over caused the toxic effects on splenic development, which was characterized by reducing growth index and lymphocytes in the white and red pulp histopathologically, increasing cell percentages of the G0/G1 phase and decreasing cell percentages of the S phase, and reducing T cells and B cells as well as IgA, IgG, and IgM contents when compared with those in the control group. Concurrently, cytokines including interleukin-2 (IL-2), transforming growth factor beta (TGF-β), tumor necrosis factor alpha (TNF-α), interferon gamma (IFN-γ) and cyclin (E/D and CDK2/4) protein expression levels were markedly decreased (*P* < 0.05 or *P* < 0.01), and interleukin-10 (IL-10) protein expression levels were significantly increased (*P* < 0.05 and *P* < 0.01) in the three NaF-treated groups. Toxic effects finally impaired the splenic cellular immunity and humoral immunity due to the reduction of T and B cell population and activity. Cell cycle arrest is the molecular basis of NaF-caused toxic effects on the splenic development.

## INTRODUCTION

Fluorine is abundant in the environment and its relationship to human health is quite extensive and spans a wide variety of fields including medicine, dentistry, environmental, toxicology, geochemistry and so on [1]. Fluoride is essential for normal maintenance of teeth, bones and has many roles in medicinal chemistry [2–4]. Fluoride is a naturally occurring contaminant in the water and most fluorosis is derived from drinking water [5]. Besides drinking water, fluorine can enter the body through food, dental products, drugs and industrial emission [1, 4]. Prolonged exposure to high concentration of fluoride is deleterious to teeth, bones, and other organs and systems [6]. As a global public health problem, fluorosis represents a severe hazard to human health, which occurs on all continents and affects millions of people [7].

Excessive intake of the fluorine induces pathological changes and disturbs the functions of many tissues and cells [8–10]. Our previous studies have documented fluorine-induced oxidative damage and pathological injury in the liver [11], kidney [12, 13], and intestine [14–16] of broiler chickens. Other studies have also shown the fluoride-caused cytotoxicity, apoptosis and DNA damage in human and animals [17–21]. In recent years, more and more studies focus on the relationship between fluoride and immunity. Our findings have recently shown that sodium fluoride (NaF) inhibits cell proliferation and induces cell apoptosis in cultured splenic lymphocytes from mice [22, 23]. It has been reported that high fluorine may affect the inflammation autoregulatory processes and the function of immune system [24]. In our previous studies, high fluorine has been found to affect immune organs including thymus [25], spleen [26–28], bursa of Fabricius [29] and cecal tonsil [30–33] by reducing the lymphocyte population and cytokine secretion, inhibiting organ growth, and causing lesions, oxidative stress and apoptosis. Excessive fluoride ingestion seriously damaged the immune function and induced thymic apoptosis in female rats [34]. Giri et al. [35] has reported that prolonged exposure to fluoride-contaminated drinking water is likely to result in immunotoxicity. Sosroseno et al. [36] has found that high concentration of NaF may be toxic to the spleen *in vitro*.

According to above-mentioned researches on effect of fluoride on immune function, very limited studies focus on the toxic effect of NaF on splenic development of human and animals *in vivo* at present. Based on our *in vitro* studies on splenic lymphocytes [22, 23], this *in vivo* study was designed to evaluate the toxic effects of NaF on splenic development, which directly provides new experimental evidence for preventing fluorosis. Thus, in the present study, different concentrations of NaF were used to investigate the splenic growth index (GI), histopathological lesions, T-cell and B-cell percentages, immunoglobulin A (IgA), immunoglobulin G (IgG) and immunoglobulin M (IgM) contents, cytokines including interleukin-2 (IL-2), interleukin-10 (IL-10), transforming growth factor beta (TGF-β), tumor necrosis factor alpha (TNF-α), and interferon gamma (IFN-γ) protein expression levels, and cell cycle and cyclins by using pathology, flow cytometry (FCM), western blot (WB) and enzyme-linked immunosorbent assay (ELISA).

## RESULTS

### Changes of growth index in the spleen

**Figure 1:**
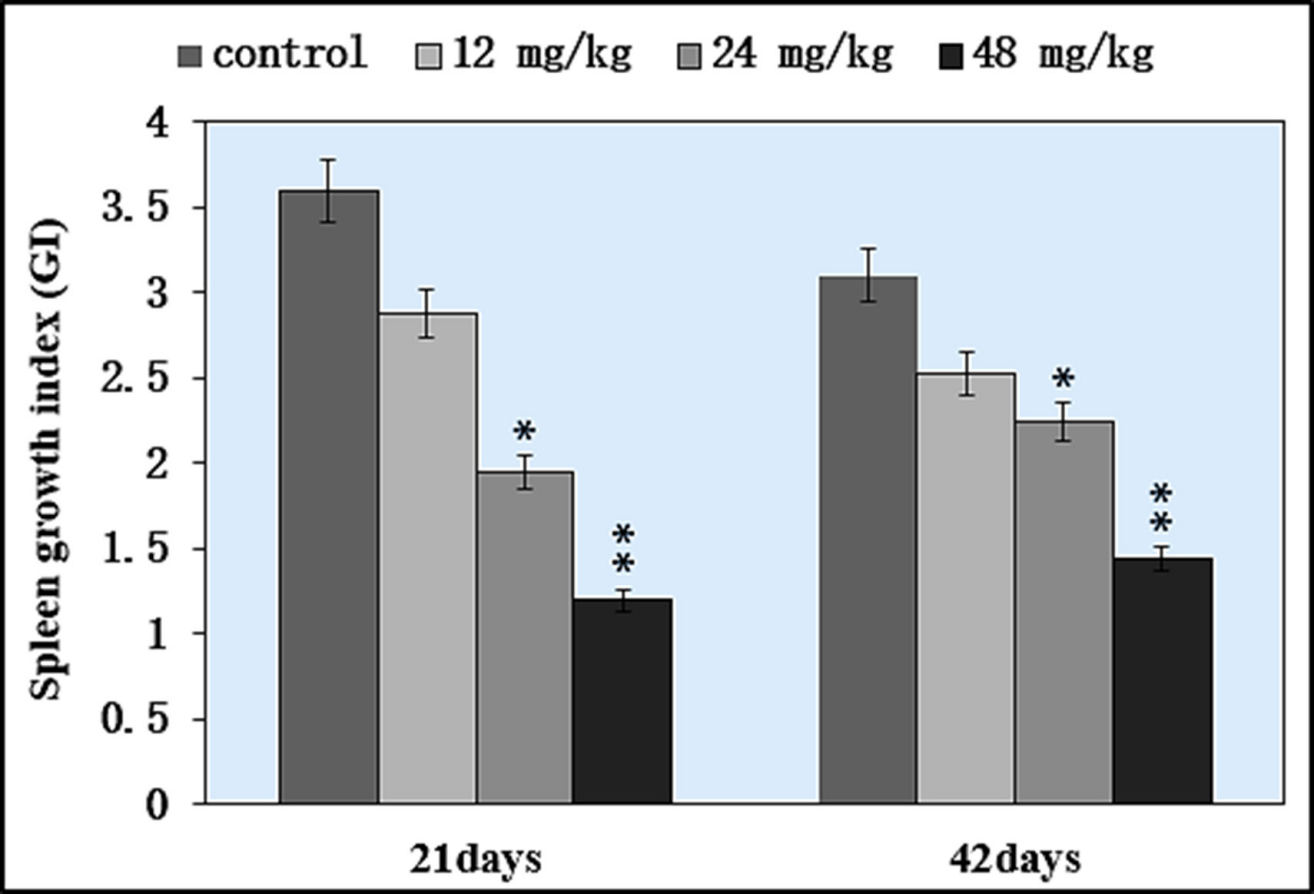
Changes of the growth index (GI) in the spleen *P < 0.05, compared with the control group; **P < 0.01, compared with the control group.

The splenic growth index (GI) was used to judge the splenic development. The results showed that there were no significant differences between the control group and the 12 mg/kg group during the 42-day experiment. However, GI was significantly lower (*P* < 0.05 and *P* < 0.01) in the 24 and 48 mg/kg groups than in the control group at 21 and 42 days of age, as illustrated in Figure 1.

### Changes of histopathological lesions in the spleen

Lymphocyte population in the white and red pulp was decreased with a dose- and time-dependent manner in the three fluoride groups when compared with that in the control group during 42-day experiment.

**Figure 2:**
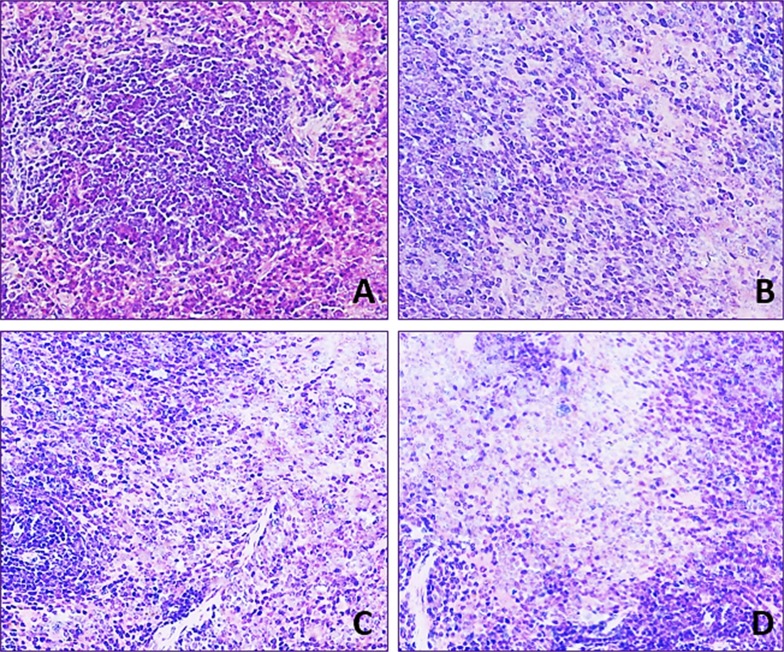
Histopathological lesions in the spleen at 21 day of age H·E × 400 Lymphocytes are decreased in the three NaF-treated groups when compared with those in the control group. The lesion degree in the order is D > C > B. (**A**) Control group. (**B**) 12 mg/kg group. (**C**) 24 mg/kg group. (**D**) 48 mg/kg group.

**Figure 3:**
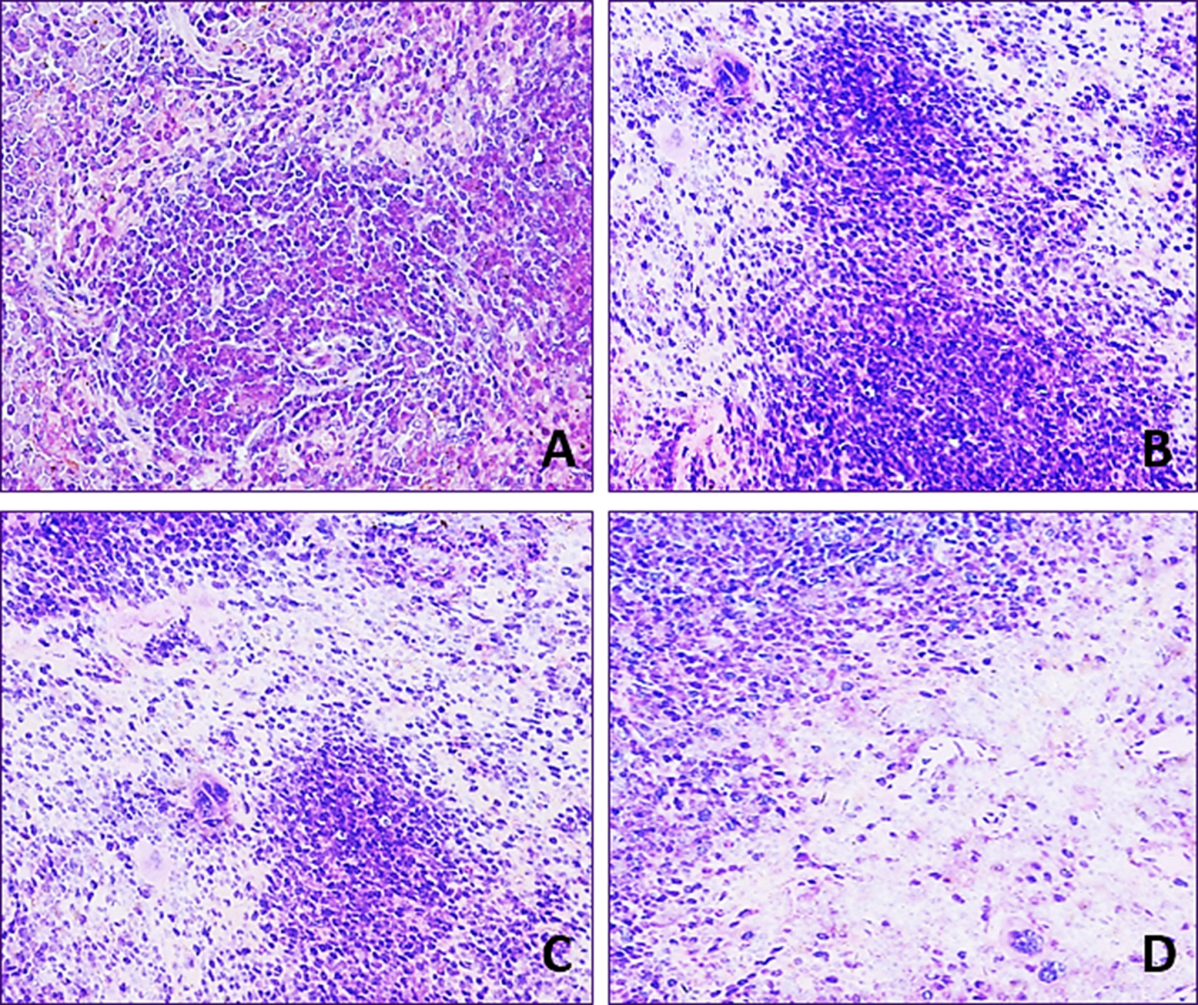
Histopathological lesions in the spleen at 42 day of age H·E × 400 Lymphocytes are decreased in the three NaF-treated groups when compared with those in the control group. The lesion degree in the order is D >C > B. (**A**) Control group. (**B**) 12 mg/kg group. (**C**) 24 mg/kg group. (**D**) 48 mg/kg group.

In 12 mg/kg group, lymphocyte numbers were decreased and the margin of white pulp began to be not clear. In 24 mg/kg group, lymphocytes were obviously decreased in the red pulp and were decreased in the white pulp. In 48 mg/kg group, the lymphocytes were significantly reduced in the white and red pulp. The results were shown in Figures 2 and 3.

### Changes of T and B-cell subsets and CD4^+^/CD8^+^ ratio in the spleen

**Figure 4:**
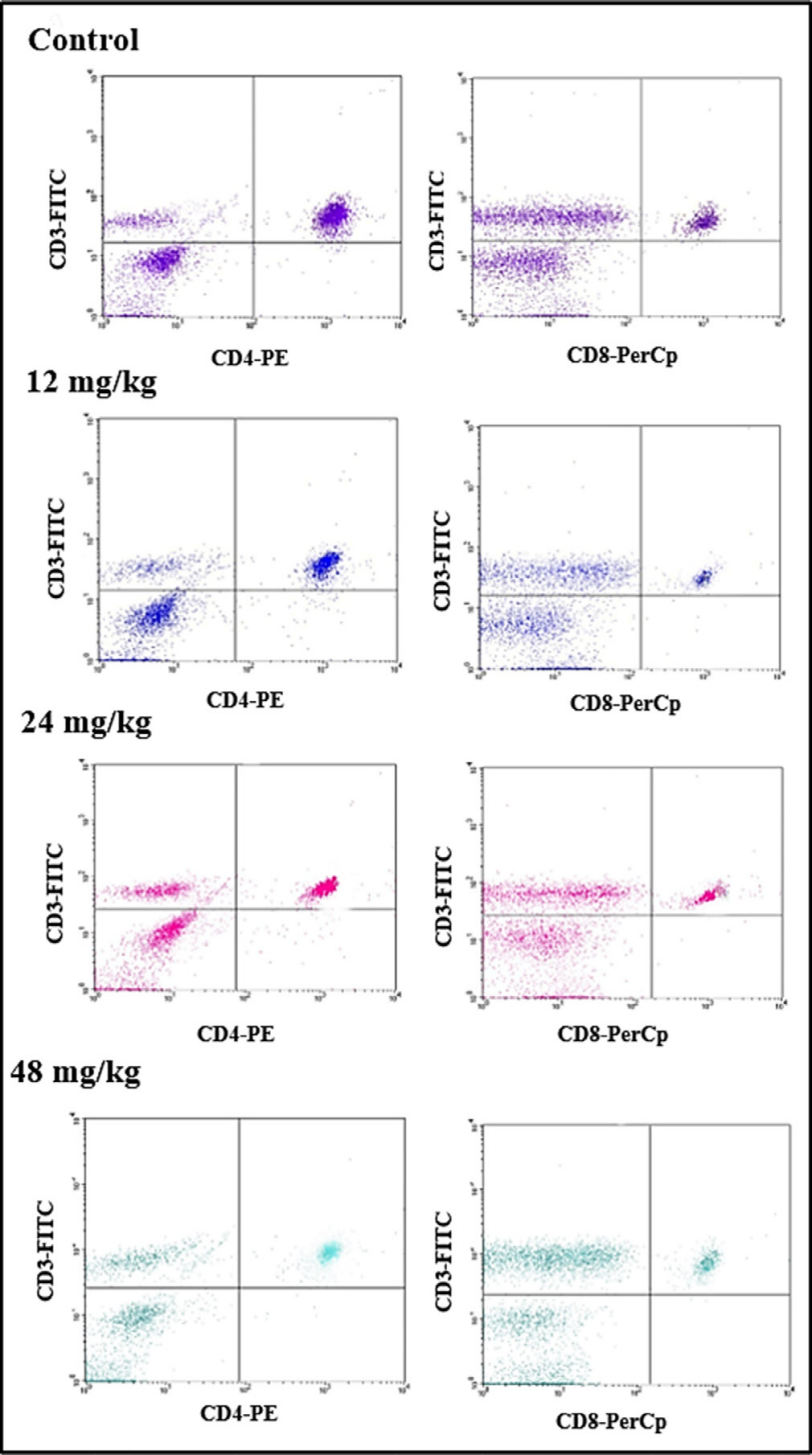
Changes of splenic T lymphocytes by FCM

**Figure 5:**
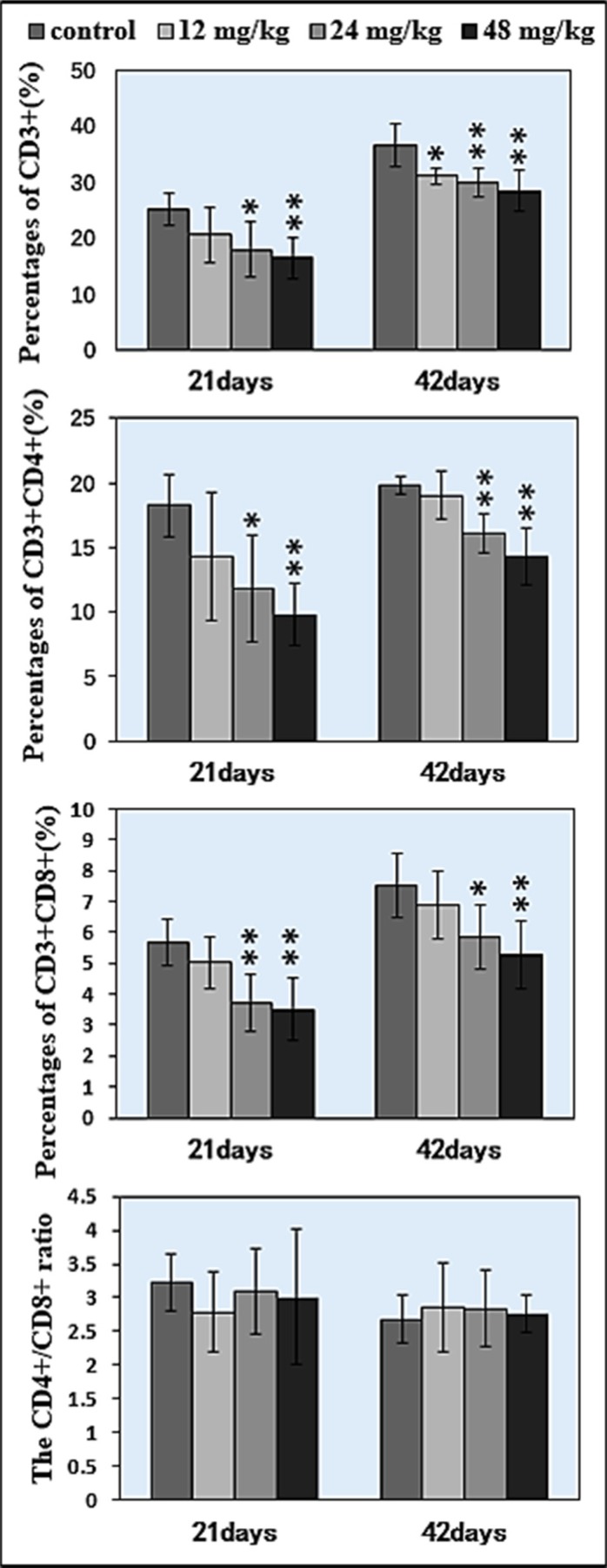
Changes of splenic T lymphocytes by FCM Changes of the percentage of CD3^+^, CD3^+^CD4^+^, CD3^+^CD8^+^ and the CD4^+^/CD8^+^ ratio. *P < 0.05, compared with the control group; **P < 0.01, compared with the control group.

The percentages of CD3*^+^*, CD3*^+^*CD4*^+^*, CD3*^+^*CD8*^+^* T lymphocytes and CD19*^+^* B lymphocytes in 21 days and 42 days were detected by FCM. The results were as follows:

The percentages of CD3*^+^* T cells were significantly decreased when compared with those in the control group. At 21 days of age, CD3*^+^* T cells were significantly lower (*P* < 0.05 and *P* <0.01) in the 24 mg/kg and 48 mg/kg NaF-treated groups than those in the control group. At 42 days of age, the percentages of CD3*^+^* T lymphocytes were significantly decreased (*P* < 0.05 and *P* < 0.01) in the 12 mg/kg, 24 mg/kg and 48 mg/kg NaF-treated groups when compared with those in the control group. And, percentages of CD3*^+^*CD4*^+^* and CD3*^+^*CD8*^+^* were significantly lower (*P* < 0.05 and *P* < 0.01) in the 24 mg/kg and 48 mg/kg NaF-treated groups than in the control group at 21 and 42 days of age. However, there were no significant changes in the CD4^+^/CD8^+^ ratios. The results were shown in Figures 4 and 5.

**Figure 6:**
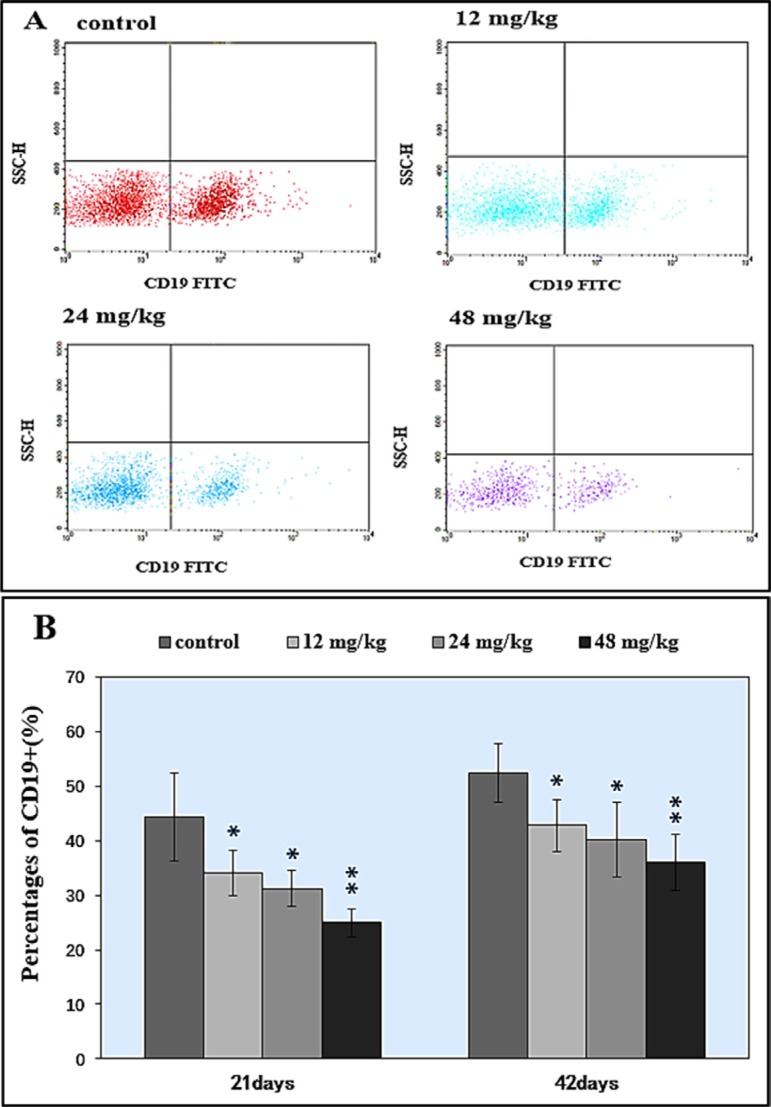
Changes of splenic CD 19^+^ B lymphocytes by FCM (**A**) Diagram of CD19^+^ analysis by FCM. (**B**) Changes of the percentages of CD19^+^.*P < 0.05, compared with the control group; **P < 0.01, compared with the control group.

In Figure 6A–6B, percentages of CD19*^+^* B lymphocytes were significantly decreased (*P* < 0.05 and *P* < 0.01) in the 12, 24 and 48 mg/kg NaF-treated groups in comparison with those in the control group at both 21 and 42 days of age.

### Changes of cytokine expression levels in the spleen

**Figure 7:**
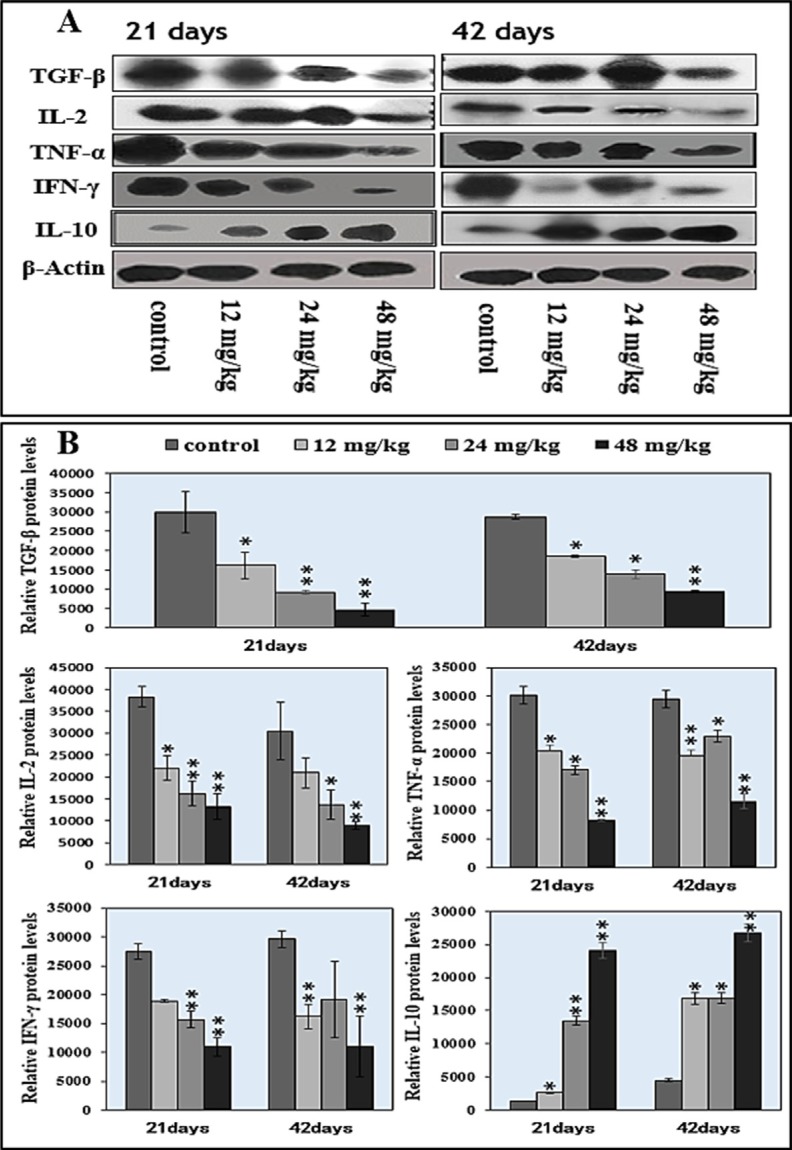
Changes of splenic cytokine protein expression levels by western blot (**A**) Western blot assay. (**B**) Quantitative measurement of the relative protein expression of IL-2, TGF-β, TNF-α, IFN-γ, and IL-10.*P < 0.05, compared with the control group; **P < 0.01, compared with the control group.

Changes of the TGF-β, TNF-α, IFN-γ, IL-2 and IL-10 protein expression levels were detected by WB, which were shown in Figure 7A. The protein expression levels of TGF-β, TNF-α, and IL-2 were significantly decreased (*P* < 0.05 or *P* < 0.01) in the 12 mg/kg, 24 mg/kg and 48 mg/kg NaF-treated groups at 21 days and 42 days of age when compared with those in the control group, as shown in Figure 7B. The IFN-γ protein expression levels were significantly lower (*P* < 0.01) in 24 and 48 mg/kg groups at 21 days of age, and in 12, 48 mg/kg groups at 42 days of age than those in the control group. However, the IL-10 protein expression levels were significantly higher (*P* < 0.05 and *P* < 0.01) in the 12 mg/kg, 24 mg/kg and 48 mg/kg NaF-treated groups than those in the control group at 21 days and 42 days of age. The results were shown in Figure 7A–7B.

### Changes of IgA, IgG, and IgM contents in the spleen

**Figure 8:**
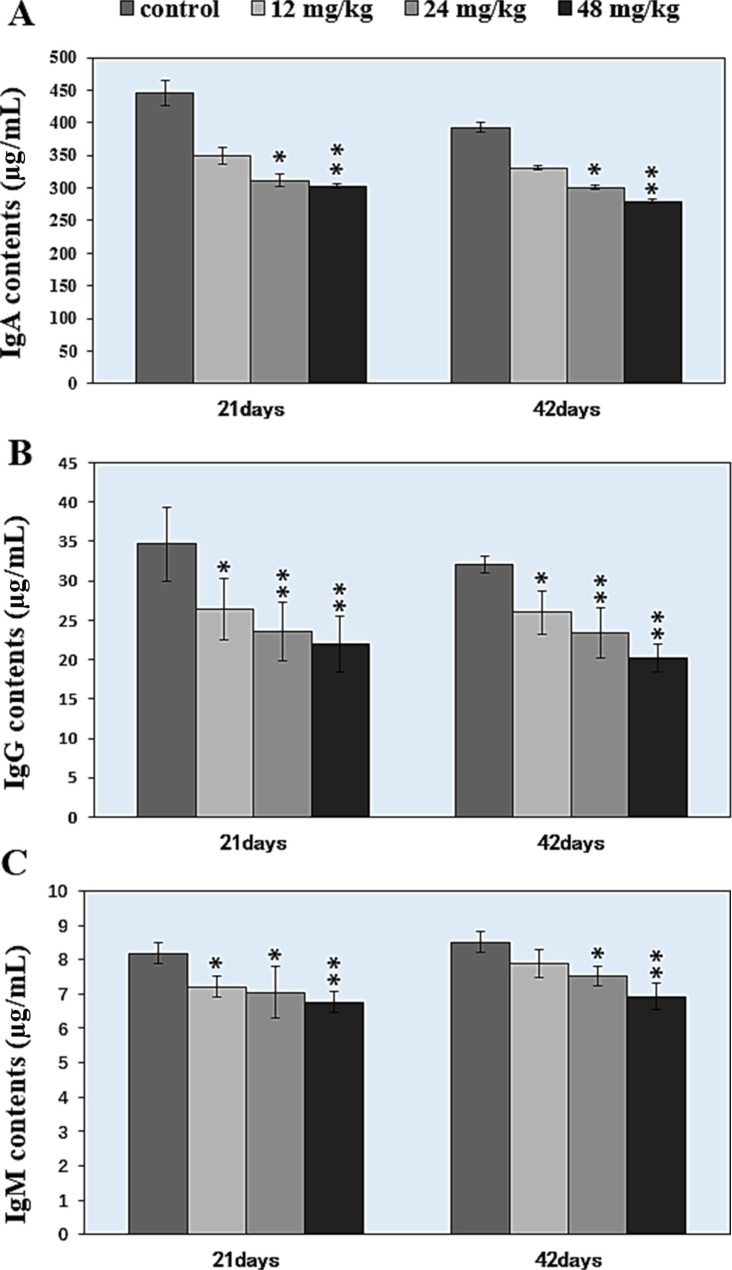
Changes of the IgA, IgG, IgM contents in the spleen *P < 0.05, compared with the control group; **P < 0.01, compared with the control group.

Changes of IgA, IgG, and IgM contents were shown in Figure 8A–8C. Figure 8A showed that the IgA contents were significantly decreased (*P* < 0.05 and *P* < 0.01) in the 24 mg/kg and 48 mg/kg NaF-treated groups at 21 days and 42 days of age when compared with those in the control group. IgG contents were significantly lower (*P* < 0.05 and *P* < 0.01) in the 12 mg/kg, 24 mg/kg and 48 mg/kg NaF-treated groups from 21 to 42 days of age than those in the control group, as shown in Figure 8B. IgM contents were significantly decreased (*P* < 0.05 or *P* < 0.01) in the 12 mg/kg, 24 mg/kg and 48 mg/kg NaF-treated groups at 21 days of age and in the 24 mg/kg and 48 mg/kg NaF-treated groups at 42 days of age in comparison with those in the control group (Figure 8C).

### Changes of cell cycle in the spleen

**Figure 9:**
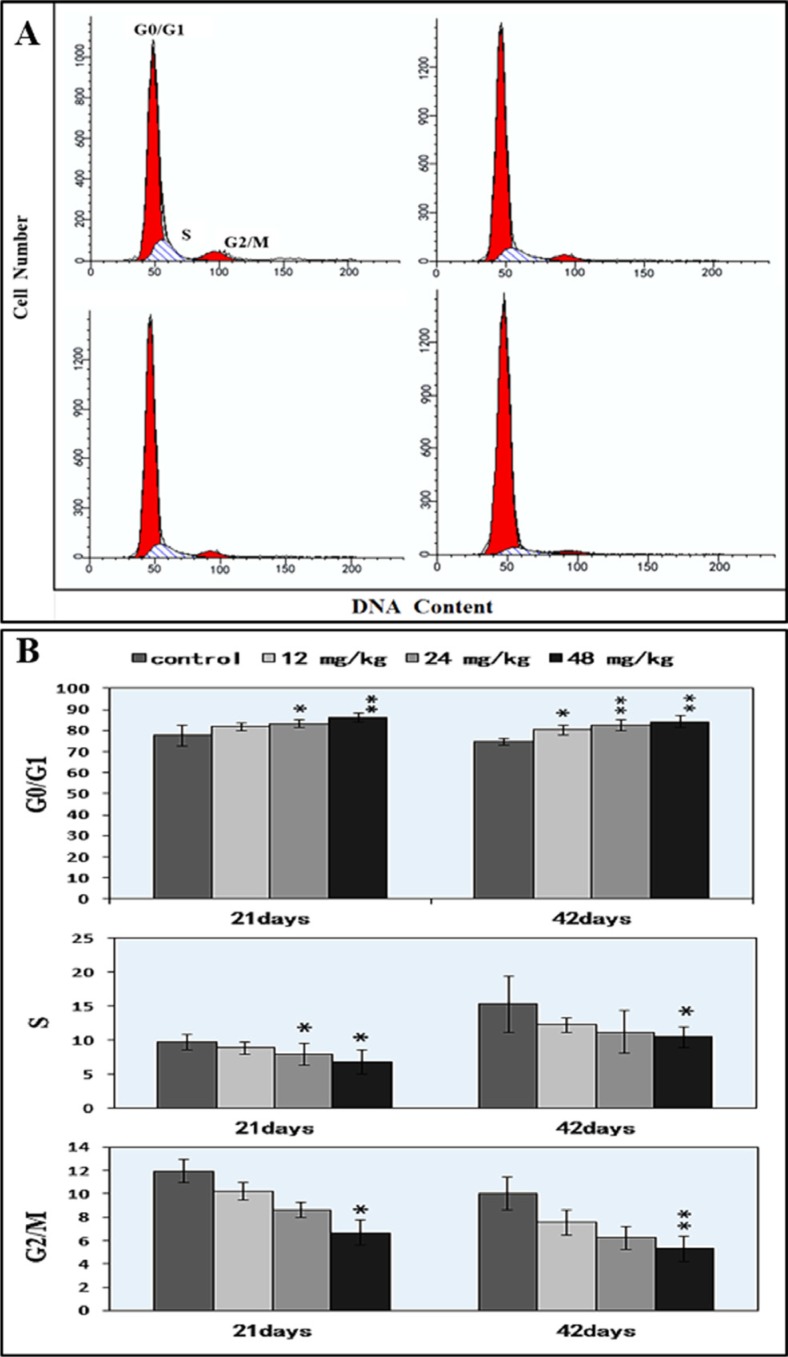
Changes of the splenic cell cycle by FCM (**A**) Diagram of cell cycle analysis by FCM. (**B**) Changes of the percentage of G0/G1, G2/M, and S phase.*P < 0.05, compared with the control group; **P < 0.01, compared with the control group.

As illustrated in Figure 9A–9B, NaF inhibited DNA synthesis of splenocytes in a dose-dependent manner. At 21 days of age, 24 mg/kg and 48 mg/kg NaF treatment significantly increased (*P* < 0.05 and *P* < 0.01) the cell percentages in the G0/G1 phase and decreased (*P* < 0.05) the cell percentages in the S and G2/M phases. At 42 days of age, the cell percentages in the G0/G1 phase were significantly increased (*P* < 0.05 and *P* < 0.01) in the 12 mg/kg, 24 mg/kg and 48 mg/kg NaF-treated groups when compared with those in control group. The cell percentages in the S phase and G2/M phases were lower (*P* < 0.05 or *P* < 0.01) in the 48 mg/kg NaF-treated group than those in the control group.

### Changes of cyclins/cdks protein expression levels in the spleen

**Figure 10:**
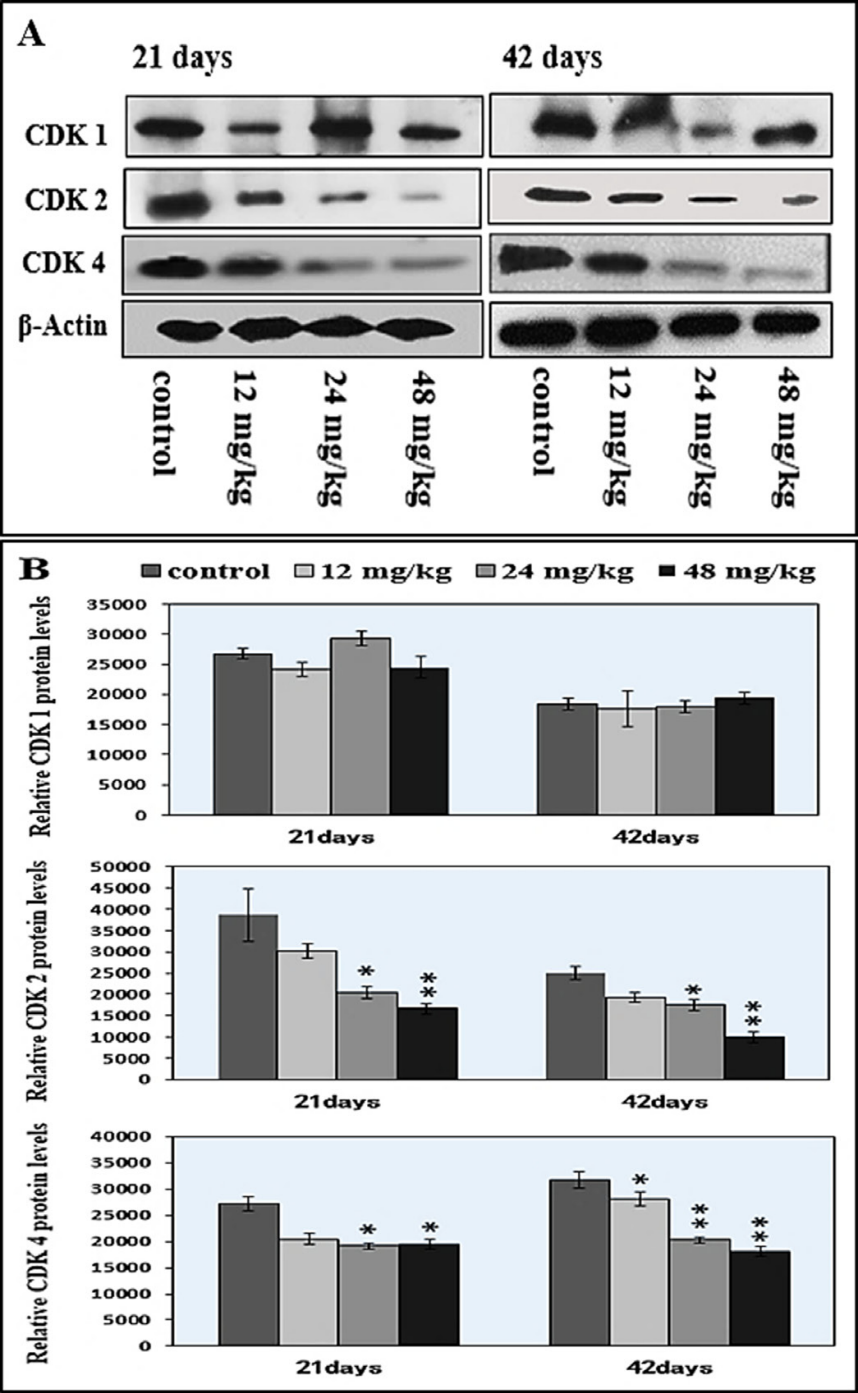
Changes of cdk1/2/4 protein expression levels in the spleen by western blot (**A**) Western blot assay. (**B**) Quantitative measurement of the relative protein expression of CDK1, CDK2 and CDK4.*P < 0.05, compared with the control group; **P < 0.01, compared with the control group.

**Figure 11:**
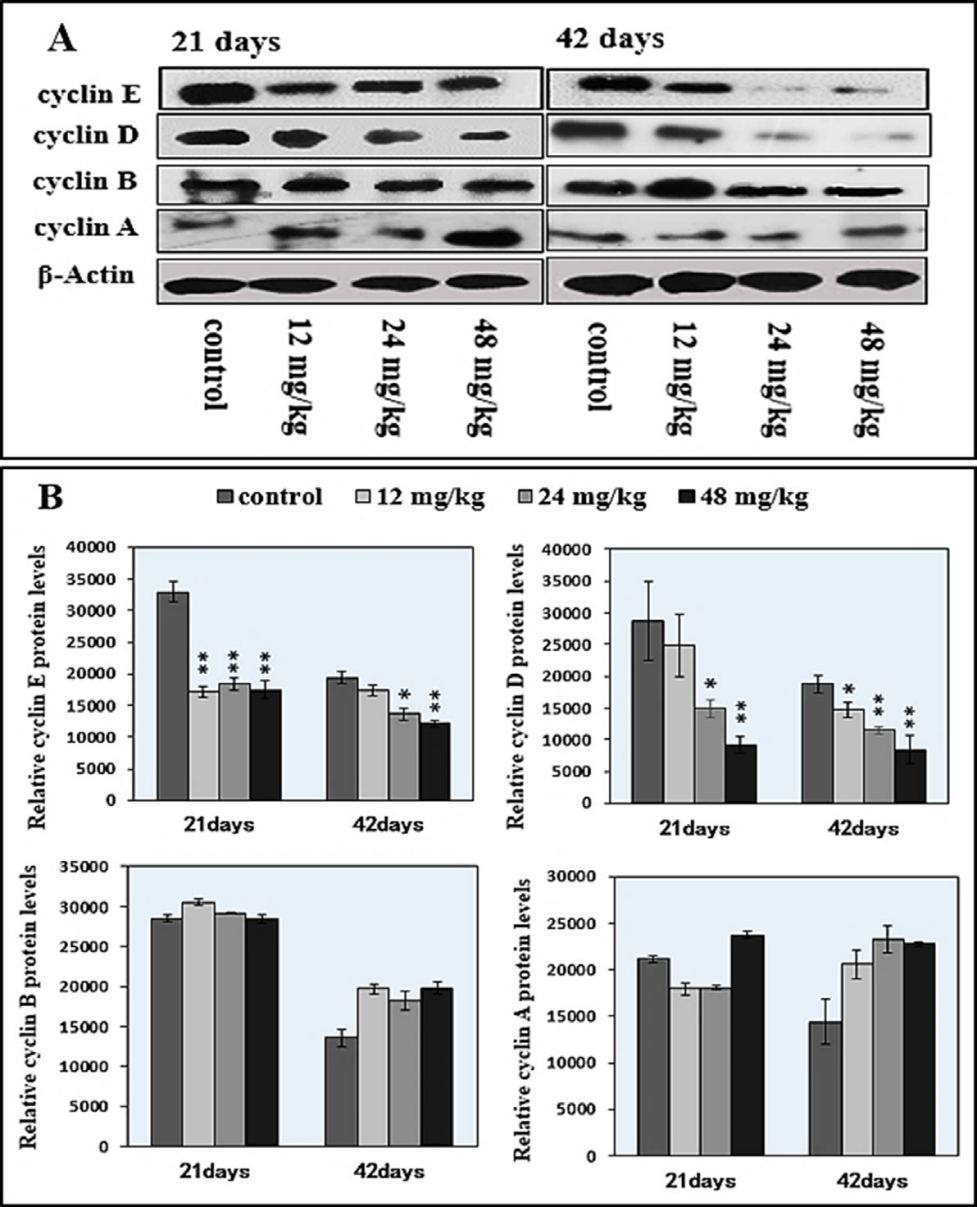
Changes of cyclin E/D/B/A protein expression levels in the spleen by western blot (**A**) Western blot assay. (**B**) Quantitative measurement of the relative protein expression of cyclin E, cyclin D, cyclin B and cyclin A. *P < 0.05, compared with the control group; **P < 0.01, compared with the control group.

As G1 phase-related regulatory molecules, cyclin E/D/B/A and cdk 1/2/4 protein expression levels were detected to reveal the mechanism of NaF-arrested G1 phase of the cell cycle. The results were shown in Figures 10, 11.

In Figure 10A–10B, the protein expression levels of CDK 2 and CDK 4 were significantly decreased (*P* < 0.05 and *P* < 0.01) in the 24 mg/kg and 48 mg/kg NaF-treated groups from 21 to 42 days of age, and the CDK 4 protein expression level was decreased (*P* < 0.05) in the 12 mg/kg NaF-treated group at 42 days of age when compared with those in the control group. However, there were no significant changes in the CDK 1 protein expression levels.

Figure 11A–11B showed that the 24 mg/kg and 48 mg/kg NaF treatment significantly reduced (*P* < 0.05 or *P* < 0.01) cyclin D and E protein expression levels at 21 and 42 days of age in comparison with those in the control group. Additionally, cyclin E protein expression levels at 21 days of age and cyclin D protein expression levels at 42 days of age were significantly decreased (*P* < 0.05 or *P* < 0.01) in the 12 mg/kg NaF-treated group. There were no significant effects of NaF treatment on cyclin A and cyclin B.

## DISCUSSION

This study was firstly conducted to define the toxic effects of NaF on splenic development *in vivo*. Indeed, NaF in 12–48 mg/kg was found to have toxic effects on spleen, including histopathological lesions, reduced relative weight, arrested cell cycle, decreased T cells and B cells as well as immunoglobulin contents in mice.

The spleen is the largest secondary lymphoid organ which contains about one-fourth of the body's lymphocytes, and initiates immune responses to blood-borne antigens [37], and plays crucial roles in responding to immune stimuli, producing immunoglobulin and cytokines, and promoting cell differentiation [38]. Das et al. [39] has reported that chronic exposure to fluoride likely results in immunotoxicity and damages spleen. Also, Peng et al. [40] has reported that fluorine ion decreases cultured splenic CD3*^+^* T lymphocytes of male Kunming mice *in vitro*. In our previous study *in vitro*, NaF has been also confirmed to decrease the percentages of splenic T and B lymphocytes of mice *in vitro* [22]. In this study, we found that NaF in excess of 12 mg/kg significantly reduced the percentages of CD3*^+^*, CD3*^+^*CD4+, CD3*^+^*CD8*^+^* T lymphocytes (*P* < 0.05 and *P* < 0.01) in the spleen (Figures 4 and 5). Similarly, the percentages of CD19*^+^* B lymphocytes were significant lower (*P* < 0.05 and *P* < 0.01) in the three NaF-treated groups than those in the control group (Figure 6A–6B). Those findings were consistent with the results of Liu et al. [31, 32], and Luo et al. [15] that dietary high fluorine reduced the numbers of T and B lymphocytes in the cecal tonsil, and B lymphocytes in the intestinal mucous [41] of broiler chickens. At present, there are no reports on the NaF-decreased splenic T cells and B cells *in vivo*. Decrease in the splenic T cells and B cells numbers and activities finally reduces splenic cellular immunity and humoral immunity. Additionally, decrease in the splenic T cells and B cells numbers and activities is consistent with the alterations of cytokines and immunoglobulins.

There are no studies on effects of NaF on splenic cytokines *in vivo* so far. As critical regulators of the immune system, cytokines can not only impact the development and differentiation of immunocytes, but also have internal interaction [42]. IL-2, TNF-α and IFN-γ are crucial cytokines to reflect the regulation of immune function, which enhances cell-mediated immunity. In the present study, protein expression levels of IL-2, TNF-α, and IFN-γ were decreased (*P* < 0.05 or *P* < 0.01) in the three NaF-treated groups (Figure 7A–7B), indicating that NaF could reduce the population of T lymphocytes and decrease cell-mediated immune function. The reduction of IL-2, TNF-α, and IFN-γ levels were consistent with previous reports in cecal tonsil of broiler chickens fed high fluoride diets [30, 32]. Concurrently, TGF-β protein expression levels were significantly decreased (*P*<0.05 and *P* < 0.01) and IL-10 protein expression levels were significantly increased (*P* < 0.05 and *P* < 0.01) in the three NaF-treated groups (Figure 7A–7B). IL-10 and TGF-β are inhibitory cytokines and are synthesized by T cells and B cells. TGF-β can promote survival of activated T cells, and enhance Th1 cell differentiation in the presence of IFN-γ *in vitro* [43]. IL-10, as an immunosuppressive cytokine, has an essential role in the establishment of peripheral tolerance to allergens and protects the host from exaggerated inflammatory responses to pathogens as well as autoimmune diseases [44], and can also inhibit T-cell proliferation and cytokine production [45, 46]. Decreased TGF-β protein expression levels and increased IL-10 protein expression levels imply that T cell and B cell numbers and activities are decreased.

Immunoglobulins, as secreted by B cells mediate interactions between antigen molecules and a variety of cellular and humoral effectors. Thus, immunoglobulins can be used to evaluate the condition of immune system. In Figure 8, the splenic IgA, IgG and IgM contents were significantly decreased (*P* < 0.05 and *P* < 0,01) in the NaF-treated groups, which was consistent with the reduction of B lymphocytes. Our previous findings have also shown that high fluorine decreases IgA, IgG and IgM contents in the cecal tonsil [31], intestines [15] of broiler chickens. However, no findings about the NaF-reduced the splenic IgA, IgG and IgM contents *in vivo* have been reported at present. The direct reason of decreased IgA, IgG and IgM contents is the reduction of B lymphocyte population and activity. Splenic humoral immune function in mice is finally impaired due to the reduction of B lymphocytes and Immunoglobulins.

It has been reported that NaF can inhibit DNA synthesis, and induce DNA damage, cell-cycle arrest in cultured rat hippocampal neurons [47, 48]. In order to reveal how NaF reduced splenic T cells and B cells, we used FCM to measure the cell cycle of splenocytes. The results showed that NaF caused G1 phase cell-cycle arrest with significantly increase (*P* < 0.05 and *P* < 0.01) in the cell percentages of G0/G1 phase (Figure 9A), which was supported by the NaF-decreased splenocyte numbers in the S phase. The cell-cycle arrest in the G0/G1 phase inhibited damaged cells to stop DNA replication at G0/G1 phase. Thus, the cell-cycle arrest inhibited lymphocytes (including T cells and B cells) proliferation, which contributed to the reduction of lymphocytes in the white/red pulp (Figures 2 and 3) and splenic GI (Figure 1) in mice. In the present study, lymphocytes in the white and red pulp were histopathologically decreased with a dose- and time-dependent manner in the NaF-treated groups during 42-day experiment (Figures 2 and 3). As a satisfactory measure of nutritive value, GI can also represent the growth state of organs. In the present study, the GI was used to judge the splenic development, and was lower in the NaF-treated groups than that in control group (Figure 1), implying that NaF inhibited the splenic development, and then impaired the splenic function in mice.

NaF-caused the cell-cycle arrest in this study is consistent with the previous reports on fluoride-increased the cell proportion in G0/G1 phase in spleen [28, 49]. Progression through a specific phase of the cell cycle is under the control of a specific class of cyclins and Cdks [50]. Progression from G1 to S phase of the mammalian cell cycle is regulated by cyclin D-dependent kinases including CDK4 and CDK6 bound to D-type cyclins, and by CDK2 bound to cyclins E or A [51]. In this study, protein expression levels of cyclin E/CDK2 and cyclin D/CDK4 were significantly decreased, and protein expression levels of cyclin B, cyclin A, and CDK1 were not changed (Figures 10 and 11), which indicated that NaF treatment slowered the G1 process and blocks the G1/S transition. These results are consistent with our previous study on sodium fluoride suppressed splenic lymphocytes proliferation *in vitro* [22]. And, Ngoc et al. [21] has also reported that NaF decreases the cyclin E levels in mouse embryonic stem cells. According to abovementioned discussion, it could be defined that NaF damage DNA synthesis, arrested cell-cycle progression, inhibited cell proliferation and caused cytotoxicity in mice.

## MATERIALS AND METHODS

### Animals and treatment

240 healthy ICR mice (Experimental Animal Corporation of DOSSY at Chengdu, China) were used in this study to estimate the toxic effects of NaF on splenic development. Food and water was provided *ad libitum*. Mice were randomly divided into 4 groups (*N* = 60). The control group was given an intragastric administration of distilled water at the same time as other groups. The experimental groups were given an intragastric administration of 12, 24, and 48 mg/kg NaF (Chengdu Kelong Chemical Co., Ltd., Chengdu, China), respectively. The gavage doses of four groups were 1 mL/100 g body weight once daily for the last 42 days.

Our experiments involving the use of mice and all experimental procedures were approved by the Animal Care and Use Committee, Sichuan Agricultural University.

### Determination of the growth index in the spleen

After body weights were recorded, eight mice in each group were euthanasia at 21 days and 42 days of age. Macroscopic observation and weight of the spleen from each mouse were recorded. The splenic growth index (GI) was calculated by the following formula:

$$GI=\frac{\text{organ weight}(\text{mg})}{\text{body weiht}(g)}$$

### Observation of histopathological lesions in the spleen

At 21 and 42 days of age, splenic samples of eight mice in each group were taken and fixed in 4% paraformaldehyde solution, and embedded in paraffin. Slides were stained with hematoxylin and eosin (H·E) for histopathological examination under a light microscope.

### Determination of T-cell and B-cell subsets in the spleen by FCM

At 21 and 42 days of age, splenic samples of eight mice in each group were taken to determine the percentages of CD3*^+^*, CD4*^+^*, CD8*^+^* T lymphocyte and CD 19*^+^* B lymphocyte by FCM.

Each spleen was cut into pieces and then filtered through nylon gauze as splenic single-cell suspension. The suspension was centrifuged at 200 × g for 5 min. The supernatant was discarded and lymphocytes were collected. The cell concentration was determined by using the normal counting method of blood cells and then diluted to 1.0 × 10*^6^* cells/mL with phosphate-buffered saline (PBS). A total of 100 μL cell suspensions was transferred to another centrifuge tube. The cells were respectively stained with 10 μL hamster anti-mouse CD3e-FITC (BD, Cat No: 553062), rat anti-mouse CD4-PE (BD, Cat No: 557308), rat anti-mouse CD8a-PerCP (BD, Cat No: 553036) and FITC anti-mouse CD19*^+^*(BD, Cat No: 553785) for 30 min at RT, and then 2 mL PBS was added and centrifuged at 200 × g for 5 min. The supernatant was discarded. Cells were resuspended in 0.5 mL PBS and determined by BD FACS Calibur flow cytometer.

### Determination of splenic cytokines by western blot

At 21 and 42 days of age, splenic samples of eight mice in each group were taken to determine the cytokine protein expression levels by western blot.

The spleen was ground into homogenate and then proteins were extracted with RIPA lysis buffer and kept in laemmli loading buffer. Protein samples were resolved on SDS-PAGE (10%–15% gels) and transferred to nitrocellulose filter membranes. Membranes were blocked with 5% fat-free milk for 1h and incubated with primary antibodies overnight at 4°C. The primary antibodies were TNF-α, IFN-γ, TGF-β, and IL-2, IL-10 (Santa Cruz, USA). The membranes were then washed with PBS-tween and incubated with biotin-conjugated secondary antibodies (Santa cruz, USA) for 1h, and washed again with PBS-tween. Blots were visualized by ECL^TM^ (Bio-Rad, Hercules, CA, USA) and X-ray film.

### Determination of splenic IgA, IgG, and IgM contents by ELISA

At 21 and 42 days of age, splenic samples were taken from mice. The spleen was weighed and homogenized in nine volumes of chilled 0.85 % NaCl solution in a chilled homogenizer and then centrifuged at 3,000 × g for 10 min at 4°C immediately. The supernatant was conserved for future analysis. Contents of the spleen IgA, IgG, and IgM were determined by using ELISA as described by Gaca et al. [52]. Those immunoglobulin contents in the spleen were quantified by using the IgA kit (Nanjing Jiancheng Bioengineering Institute, Cat No: H108), IgG kit (Nanjing Jiancheng Bioengineering Institute, Cat No: H106), IgM kit (Nanjing Jiancheng Bioengineering Institute, Cat No: H109). The IgA, IgG, and IgM contents were determined by the standard curve and expressed as micrograms per milliliter.

### Determination of splenic cell cycle by FCM and western blot

At 21 and 42 days of age, spleen samples of eight mice in each group were taken to determine the cell cycle by FCM.

Pretreatment methods of splenic single-cell suspension were the same as above descripted splenic T and B cell subsets. Then, splenic cells were incubated for 30 min at room temperature in the dark with 0.25% Triton X-100 and 5 μL propidium iodide (PI) (BD, Cat. No.51–66211E, USA ). Cells were resuspended in 0.5 mL PBS and determined by BD FACS Calibur flow cytometer. The results were analyzed by the use of the Mod Fit LT for Mac V3.0 computer program.

At the same time, the cyclins/cdks protein expression levels were determined by WB at 21 and 42 days of age. Pretreatment methods of splenic proteins extraction, SDS-PAGE, immunoreaction and experimental operation of WB were the same as above descripted splenic cytokine detection. The primary antibodies were cyclin D/E/B/A, cdk1/2/4 (Abcam, UK).

### Statistical analysis

The experimental data are expressed as the mean ± standard deviation. One-way analysis of variance (ANOVA) procedure in SPSS 17.0 software was used to assess statistical significances between F-treated group and control group. A value of *P* < 0.05 was considered significant, and *P* < 0.01 was markedly significant.

## CONCLUSIONS

According to the results in this study and above-mentioned discussion, it is concluded that NaF in 12 mg/kg and over causes toxic effects on the splenic development *in vivo* by decreasing GI, causing histopathological lesions, arresting cell cycle, and reducing T cells and B cells as well as immunoglobulin contents in mice. Cell cycle arrest is the molecular basis of NaF-caused toxic effects on the splenic development. Toxic effects on the splenic development finally impair cellular and humoral immunity due to the reduction of T, B cell numbers and activities.
